# Soil legacy effects of long-term nitrogen addition on litter decomposition and soil CO_2_ efflux in a subtropical grassland: a short-term incubation study

**DOI:** 10.3389/fpls.2026.1849132

**Published:** 2026-06-15

**Authors:** Chang Liu, Ruifen Zhu, Bo Yao, Jishan Chen

**Affiliations:** 1 Chongqing Academy of Animal Science, Chongqing, China; 2 Pratacultural Engineering and Technology Research Center of Chongqing, Chongqing, China

**Keywords:** carbon cycling, CO2 efflux, litter decomposition, litter quality, nitrogen addition, soil legacy effects, subtropical grassland

## Abstract

Litter decomposition is a key process in carbon (C) and nutrient cycling, but how nitrogen (N) addition affects decomposition via soil properties versus altered litter quality remains unclear. We conducted a 30-day laboratory incubation using litter from three grassland species (*Poa annua*, *Lolium perenne*, *Dactylis glomerata*) and soils from a four-year N addition field experiment (0, 1.49, 2.99, 5.99 g N·m^-2^·yr^-1^) in a subtropical grassland of Southwest China. Because all litter was collected from unfertilized areas, our experiment specifically tested the soil legacy effects of N addition on decomposition of standardized litter, not the direct effects on litter quality. Decomposition rates (decay constant k) ranged from 0.017 day^-1^ (*P. annua*, no N) to 0.068 day^-1^ (*L. perenne*, high N). N addition significantly increased decomposition for all species, with the strongest stimulation for low-quality litter (*P. annua*). First-order kinetic models fitted the 30-day data well (R² > 0.98), but extrapolation beyond the incubation period is not justified. Initial C:N ratio was negatively correlated with both k (*r* = –0.625, *P* < 0.01) and cumulative CO_2_ efflux significantly influenced by both species identity and N addition. Our design does not allow attribution of the stimulation to N-induced changes in litter quality. Instead, long-term N addition altered soil properties (increased inorganic N, reduced pH), which in turn modulated decomposition of subsequently added litter. We conclude that soil legacy effects of N addition accelerate litter decomposition and CO_2_ efflux, with effects modulated by litter quality. These findings provide empirical evidence for predicting ecosystem C dynamics under future N deposition but highlight the need for longer-term experiments that directly manipulate both soil properties and litter chemistry.

## 1 Introduction

Plant litter decomposition represents one of the most fundamental ecological processes in terrestrial ecosystems, serving as the primary pathway for the return of carbon (C) and essential nutrients from vegetation to soils (Berg and McClaugherty, 2014; Prescott, 2010). Approximately 50-70% of annual net primary production returns to soils through litter decomposition (Gessner et al., 2010). Grasslands cover approximately 40% of the Earth’s terrestrial surface and store about 20% of global soil organic carbon, rendering them highly sensitive to environmental changes (Bai et al., 2010; Conant et al., 2001). Subtropical grasslands, in particular, are characterized by rapid nutrient cycling rates, making them especially vulnerable to perturbations in C dynamics (Song et al., 2011).

Litter decomposition is governed by three primary controls: climate, litter quality, and decomposer communities (Aerts, 1997). Among these, litter quality—typically characterized by initial nitrogen (N) content and carbon-to-nitrogen (C:N) ratio—is widely recognized as a key determinant of decomposition rates at local to regional scales (Melillo et al., 1982; Cornwell et al., 2008). High-quality litter generally decomposes more rapidly than low-quality litter because it provides more readily available nutrients for decomposer microorganisms (Hobbie, 2008; Makkonen et al., 2012).

The past century has witnessed a dramatic increase in anthropogenic N deposition globally, with China emerging as a global hotspot (Liu et al., 2013; Galloway et al., 2008). However, the effects of N addition on litter decomposition remain highly variable and context-dependent (Knorr et al., 2005; Peng et al., 2022; Zhang et al., 2019). Some studies report accelerated decomposition under N addition due to alleviation of microbial N limitation (Carreiro et al., 2000), while others document decelerated decomposition, particularly for lignin-rich litter (Fog, 1988). Meta-analyses have further shown that the direction and magnitude of N addition effects depend on litter quality, ecosystem type, and experimental duration (Zhou et al., 2022; Wang et al., 2025). These inconsistent findings highlight the need for targeted investigations that explicitly consider the interactive effects of N addition and litter quality.

In N-limited grasslands, where decomposer communities are often constrained by N availability, N addition may exert particularly strong effects on decomposition processes (Song et al., 2011; LeBauer and Treseder, 2008). Recent studies have emphasized that long-term N deposition can create soil legacy effects that persist for years, influencing carbon cycling through changes in microbial communities and soil physicochemical properties (Kou et al., 2023; Stuble et al., 2024). However, few studies have systematically examined how different levels of N addition interact with litter from coexisting plant species to influence decomposition rates and soil CO_2_ efflux in subtropical grasslands. Moreover, the potential for N addition to alter plant community composition—such as the loss of N-fixing legumes—represents an indirect pathway through which N deposition may modify litter inputs and decomposition dynamics over longer timescales (Lü et al., 2023; Bai et al., 2010).

Soil CO_2_ efflux, which integrate autotrophic root respiration and heterotrophic microbial decomposition, represent the largest terrestrial C flux to the atmosphere (Raich and Schlesinger, 1992; Bond-Lamberty and Thomson, 2010). The priming effect—where fresh organic matter inputs stimulate decomposition of native soil organic matter—can further amplify CO_2_ efflux in response to litter inputs (Kuzyakov, 2002; Fontaine et al., 2004). Understanding how N addition modulates these processes is critical, especially given that microbial carbon use efficiency and necromass turnover play key roles in soil carbon stabilization (Liang et al., 2020; Crowther et al., 2019).

In this study, we conducted a controlled laboratory incubation experiment to investigate the soil legacy effects of long-term N addition on litter decomposition and CO_2_ efflux. We addressed three primary research questions: (1) How does initial litter quality differ among dominant grassland species, and how does this influence decomposition rates? (2) How does N addition affect decomposition rates and CO_2_ efflux, and do these effects vary with litter quality? (3) What soil properties mediate these effects? We tested two hypotheses: (1) Litter with higher initial N content and lower C:N ratio will decompose more rapidly. (2) Nitrogen addition will accelerate litter decomposition, with the magnitude of stimulation being greater for low-quality litter due to alleviation of microbial N limitation in soils with a history of N enrichment.

## 2 Materials and methods

### 2.1 Study site description

The study was conducted at the Forage Research Station of the Chongqing Academy of Animal Science in Wuxi County, Chongqing Municipality, Southwest China (108°51′E, 31°40′N; elevation 2100 m). The region experiences a typical subtropical monsoon climate, characterized by warm, humid summers and mild, relatively dry winters. Based on meteorological data from 2000 to 2020, the mean annual temperature is 17.8°C, with monthly mean temperatures ranging from 41.6 °C in July to -2.2°C in January. The mean annual precipitation is 1669.7 mm, approximately 75% of which occurs during the summer months from May through August. The mean annual accumulated temperature (≥10 °C) is 1660°C, and the frost-free period averages 172 days.

The soil at the study site is classified as yellow-brown soil according to the Chinese Soil Classification System, which corresponds to Chernozem in the FAO World Reference Base for Soil Resources. The soil is characterized by relatively low organic matter content, moderate acidity, and high permeability, typical of mountainous subtropical grassland soils. Prior to the establishment of the N addition experiment, the site was a secondary vegetation meadow with co-dominance of several grass and forb species. The dominant species included *P. annua* L., *L. perenne* L., *D. glomerata*, as well as *Trifolium repens*, *Avena fatua* L., and various *leguminous* species. Vegetative growth typically occurs from late April to early October, with canopy coverage ranging from 60% to 96% depending on seasonal conditions.

To exclude grazing disturbance, the entire study area was fenced in 2020, one year prior to the establishment of the N addition experiment. Hay was harvested once annually in September to maintain consistency in management practices. No other grassland amelioration measures (e.g., fertilization, irrigation, or reseeding) were implemented during the study period.

### 2.2 Experimental design and field N addition treatments

The N addition experiment was established in 2020 using a randomized complete block design with four N addition treatments and four replicate blocks. Each treatment plot measured 10 m × 10 m, with 2 m buffer strips between adjacent plots to minimize cross-treatment contamination. The four N addition treatments were: CK (no N addition, serving as the control), LD (low addition, 1.49 g N m^-2^y^-1^), MD (medium addition, 2.99 g N m^-2^y^-1^), and HD (high addition, 5.99 g N m^-2^y^-1^).

These N addition rates were selected to represent the range of current and projected N deposition rates in subtropical China. Regional background N deposition in the adjacent eastern margin of the Sichuan Basin has been recorded at 2.87 g N m^-2^ yr^-1^ (Wang et al., 2016), similar ranges have been reported across China (Xu et al., 2023), indicating that our LD treatment (1.49) represents a below-background level, MD (2.99) approximates ambient deposition, and HD (5.99) represents a future high-deposition scenario (Liu et al., 2013; Jia et al., 2014). Nitrogen was applied as urea (46% N), which was weighed, dissolved in 20 L of water, and applied evenly to the soil surface using a backpack sprayer. To simulate natural N deposition patterns, N was applied in two split applications: 60% of the annual total was applied in late April to early May (coinciding with the onset of the growing season), and the remaining 40% was applied in mid-June (peak growing season). Control plots received an equivalent volume of water (20 L) without urea to account for potential effects of water addition. The field treatments were maintained for four consecutive years (2020–2023) prior to soil collection.

### 2.3 Soil sampling and processing

In late September 2023, following four years of N addition, soil samples were collected from the 0–20 cm mineral soil layer in each treatment plot. Within each plot, six soil cores (2.5 cm diameter) were collected randomly, avoiding areas with obvious disturbance or concentrated animal activity. The six cores from each plot were composited to create a single representative sample per plot, yielding four replicate samples per N treatment.

Soil samples were transported to the laboratory in coolers within 6 hours of collection. Upon arrival, visible roots, soil fauna, and organic debris were carefully removed by hand. The soil was then passed through a 2 mm sieve to achieve uniformity and to remove larger organic fragments. Each composite sample was thoroughly homogenized and divided into two subsamples. One subsample was stored at 4°C for immediate processing for soil inorganic N analysis and initiation of the incubation experiment. The second subsample was air-dried at room temperature for 7 days, then ground to pass through a 0.15 mm sieve for analysis of total C, total N, total P, available P, and soil pH.

### 2.4 Litter collection and processing

Senescent (brown, fully senesced) aboveground litter was collected from three dominant plant species (*P. annua* L. (annual bluegrass, a C_4_ grass), *L. perenne* L. (perennial ryegrass, a C_3_ grass), and *D. glomerata* (orchard grass, a C_3_ grass)) present at the study site. These species were selected because they represent the dominant litter-producing species in the study area and exhibit contrasting life history strategies and leaf chemistry. Critically, all litter was collected from areas outside the N addition plots (i.e., from adjacent unfertilized areas) to avoid confounding effects of prior N exposure on initial litter chemistry. Litter collection was conducted in early October 2023, coinciding with the peak senescence period. For each species, approximately 500 g of senescent material was collected from multiple individuals across the study site to capture natural variation. The collected litter was air-dried in the field, then transported to the laboratory for further processing. In the laboratory, litter samples were dried to constant weight at 60°C for 48 hours to remove residual moisture. The dried litter was then ground to a fine powder using a ball mill (MM400, Retsch GmbH, Haan, Germany) and passed through a 0.25 mm sieve to ensure homogeneity. Each litter sample was divided into two subsamples: one for initial chemical properties (C, N, P concentrations, C:N ratio) and the other for use in the incubation experiment.

### 2.5 Laboratory incubation experiment

We conducted a 30-day laboratory incubation experiment to quantify litter decomposition rates and CO_2_ efflux under controlled conditions. The experimental design was fully factorial, combining three litter species (*Poa*, *Lolium*, *Dactylis*), four N addition treatments (CK, LD, MD, HD), and four replicate blocks, yielding 48 experimental units (3×4×4 = 48). Additionally, control incubations without litter were established for each N treatment to account for soil basal respiration (4 N treatments×4 replicates=16 control units), bringing the total number of incubation units to 64.

For each experimental unit, 0.1 g of ground litter was thoroughly mixed with 20 g of oven-dry equivalent field-moist soil (representing the corresponding N treatment) in a 500 mL glass incubation jar. Soil water content was adjusted to 60% of water holding capacity using deionized water to maintain optimal moisture conditions for microbial activity. The jars were sealed with airtight lids equipped with rubber septa for gas sampling and incubated in the dark at a constant temperature of 25°C (± 0.5°C) to eliminate temperature variability.

CO_2_ efflux were measured at 5-day intervals (days 5, 10, 15, 20, 25, and 30) using the alkali absorption method. At each sampling time, jars were opened for 30 minutes to allow air exchange, then resealed with a small vial containing 10 mL of 0.5 M NaOH solution. After 24 hours of absorption, the NaOH solution was removed and titrated with 0.1 M HCl in the presence of BaCl_2_ to determine the amount of CO_2_ absorbed. CO_2_ efflux were calculated as mg CO_2_-C per gram of litter-C input per day and cumulated over the 30-day incubation period.

To account for CO_2_ derived from soil organic matter mineralization (basal respiration), efflux from control soils (without litter) were subtracted from efflux from litter-amended soils. This approach allowed us to isolate the CO_2_ derived specifically from litter decomposition.

### 2.6 Decomposition rate modeling

Litter decomposition dynamics were described using a first-order kinetic model, which is widely used to characterize the decomposition of organic matter in soils (Olson, 1963). The model has the form:


$$C_{m}=C_{0}\left(1-e^{-kt}\right)$$

where C_m_ is cumulative CO_2_-C efflux at time t (mg CO_2_-C·g^-1^ litter-C), C_0_ is the potentially mineralizable C pool (mg CO_2_-C·g^-1^ litter-C), and k is the decomposition rate constant (day^-1^), t is incubation time (days). We acknowledge that parameter estimation from a 30-day incubation provides only a short-term approximation; extrapolation beyond the incubation period is not justified. Model parameters (C_0_) and (k) were estimated for each treatment combination by non-linear regression using the Gauss-Newton algorithm implemented in the ‘nls’ function in R (R Core Team, 2021). Model goodness-of-fit was assessed using the coefficient of determination (R^2^).

### 2.7 Chemical analyses

Total carbon (C) and nitrogen (N) concentrations in initial litter samples were determined using an elemental analyzer (Variomax CNS Analyzer, Elementar GmbH, Hanau, Germany) following the Dumas dry combustion method. Total phosphorus (P) concentration was determined using the molybdenum blue colorimetric method after wet digestion with H_2_SO_4_-H_2_O_2_ (Murphy and Riley, 1962). Litter C:N and N:P ratios were calculated on a mass basis. Soil total C and N were analyzed using the same elemental analyzer as for litter. Soil total P was determined using the sodium carbonate fusion method followed by molybdenum blue colorimetry. Available P (AP) was extracted using 0.5 M NaHCO_3_ (Olsen method) and quantified colorimetrically. Soil inorganic N (NH_4_^+^-N and NO_3_^--^N) was extracted with 2 M KCl (soil:solution ratio 1:5) and analyzed using a continuous flow analyzer (Skalar SAN, Skalar Analytical B.V., Breda, Netherlands). Soil pH was measured in a 1:2.5 soil: water suspension using a glass electrode pH meter (PHS-3C, INESA Scientific Instrument Co., Shanghai, China). All soil analyses were performed on air-dried, sieved (2 mm) samples.

### 2.8 Statistical analyses

All statistical analyses were performed using R version 4.2.1 (R Core Team, 2021). Prior to analysis, data were tested for normality using Shapiro-Wilk tests and for homogeneity of variances using Levene’s tests. Where necessary, data were log-transformed to meet assumptions of parametric tests. The effects of species identity (S), N addition (N), and their interaction on decomposition rate (at each sampling interval) and cumulative CO_2_ efflux were analyzed using two-way analysis of variance (ANOVA). When significant effects were detected, *post-hoc* comparisons were conducted using Tukey`s honestly significant difference (HSD) test. The effects of N addition on soil chemical properties (total C, total N, total P, available P, NO_3_^--^N, NH_4_^+^-N, pH) were analyzed using one-way ANOVA, followed by Tukey’s HSD test. Pearson correlation coefficients were calculated to examine relationships between initial litter quality parameters (N content, P content, C:N ratio, N:P ratio) and decomposition rates at each sampling interval, as well as cumulative CO_2_ efflux. First-order kinetic models were fitted to cumulative CO_2_ efflux data using non-linear regression, with parameters estimated via the Gauss-Newton algorithm. Model fits were evaluated using (R^2^) values. All reported values are means ± standard error (SE). Statistical significance was defined as (*P* < 0.05), with trends considered marginally significant at (0.05 ≤ *P<*0.10).

## 3 Results

### 3.1 Initial litter chemical properties

Initial litter chemical properties varied significantly among the three study species (**Table 1**). *D. glomerata* exhibited the highest N concentration (16.3 ± 1.3 mg·g^-1^), followed by *L. perenne* (15.6 ± 1.4 mg·g^-1^), and *P. annua* (12.5 ± 1.3 mg·g^-1^) (*F*_2, 9_ = 5.29, *P* = 0.013). Similarly, *D. glomerata* had the lowest C:N ratio (28.5 ± 2.9), while *P. annua* had the highest (36.8 ± 2.7) (*F*_2, 9_ = 15.33, *P* = 0.016). Total C concentrations did not differ significantly among species, ranging from 460.3 to 465.3 mg·g^-1^ (*P* > 0.05). N:P ratios also varied among species, with *D. glomerata* showing the highest N:P ratio (14.8 ± 1.3) and *L. perenne* the lowest (11.1 ± 0.9) (*F*_2, 9_ = 8.21, *P* = 0.021). These differences in initial litter quality formed the basis for evaluating species-specific decomposition responses.

**Table 1:** Mean initial *C*_litter_, *N*_litter_, *P*_litter_, *C*_litter_ to *N*_litter_ ratio, and *N*_litter_ to *P*_litter_ ratio of litter from the three species.

Species	*C*_litter_ (mg g^-1^)	*N*_litter_ (mg g^-1^)	*P*_litter_ (mg g^-1^)	*C*_litter_ to *N*_litter_ ratio	*N*_litter_ to *P*_litter_ ratio
*P. annua L.*	460.3 ± 3.6^a^	12.5 ± 1.3^c^	1.0 ± 0.05^b^	36.8 ± 2.7^a^	12.5 ± 1.1^b^
*L. perennel L.*	464.3 ± 3.5^a^	15.6 ± 1.4^ab^	1.4 ± 0.04^a^	29.8 ± 2.6^b^	11.1 ± 0.9^b^
*D. glomerata*	465.3 ± 3.8^a^	16.3 ± 1.3^a^	1.1 ± 0.06^b^	28.5 ± 2.9^bc^	14.8 ± 1.3^a^

Values within the same column with different superscript letters are significantly different at the *P* < 0.05 level. Means ±1 SE are shown.

### 3.2 Soil chemical properties after four years of N addition

Four years of continuous N addition significantly altered soil inorganic N pools and pH, but had no detectable effects on total C, total N, total P, or available P (**Table 2**). Soil NH_4_^+^-N concentrations increased progressively with N addition rate, from 4.22 ± 0.06 μg·g^-1^ in CK plots to 13.31 ± 0.03 μg·g^-1^ in HD plots (*F*_3,12_ = 11.32, *P* = 0.013). Similarly, NO_3_^--^N concentrations increased from 3.20 ± 0.26 μg·g^-1^ (CK) to 8.92 ± 0.19 μg·g^-1^ (HD) (*F*_3,12_ = 12.29, *P* = 0.011). Soil pH decreased with increasing N addition, from 8.1 ± 0.20 in CK to 7.2 ± 0.20 in HD (*F*_3,12_ = 10.38, *P* = 0.016), reflecting acidification associated with nitrification processes. In contrast, soil total C (3.56-3.60 mg·g^-1^), total N (0.28-0.33 mg·g^-1^), total P (0.10-0.15 mg·g^-1^), and available P (10.22-12.32 μg·g^-1^) did not differ significantly among N treatments (all *P* > 0.05), suggesting that N addition primarily affected inorganic N pools rather than total nutrient stocks during the four-year experimental period.

**Table 2:** Soils chemical properties of different treatments before the incubation.

Treatments	*C*_soil_ (mg g ^-1^)	*N*_soil_ (mg g ^-1^)	*P*_soil_ (mg g ^-1^)	*AP*_soil_ (*u*g g ^-1^)	*NO_3_^--^N* (*u*g g ^-1^)	*NH_4_^+^-N* (*u*g g ^-1^)	*pH* (H_2_O)
CK	3.59 ± 1.0^a^	0.32 ± 0.02^a^	0.11 ± 0.01^a^	10.22 ± 0.3^a^	3.20 ± 0.26^d^	4.22 ± 0.06^d^	8.1 ± 0.20^a^
LD	3.56 ± 1.2^a^	0.31 ± 0.01^a^	0.12 ± 0.02^a^	11.38 ± 0.4^a^	7.14 ± 0.22^c^	10.63 ± 0.04^c^	7.8 ± 0.10^b^
MD	3.60 ± 1.3^a^	0.33 ± 0.02^a^	0.15 ± 0.01^a^	12.32 ± 0.2^a^	8.36 ± 0.24^b^	12.50 ± 0.09^b^	7.6 ± 0.05^c^
HD	3.58 ± 1.1^a^	0.28 ± 0.03^a^	0.10 ± 0.02^a^	11.46 ± 0.4^a^	8.92 ± 0.19^a^	13.31 ± 0.03^a^	7.2 ± 0.20^d^

Values within the same column with different superscript letters are significantly different at the P< 0.05 level. Means ±1 SE are shown.

### 3.3 Litter decomposition dynamics

Litter decomposition, measured as CO_2_ efflux rates over the 30-day incubation period, exhibited strong species-specific patterns and was significantly influenced by N addition (**Table 3**). Across all treatments, decomposition rates were highest during the first 5–10 days of incubation, declining progressively thereafter, consistent with the rapid mineralization of labile C compounds followed by slower decomposition of more recalcitrant fractions.

**Table 3:** Two-way ANOVA of the effect of species (S) and nitrogen (N) addition on rate of litter decomposition in six incubation periods (I,II, III, IV, V and VI).

Variation	Incubation periods					
	I (5 days)	II (10 days)	III (15 days)	IV (20 days)	V (25 days)	VI (30 days)
Species (S)	**<0.001**	**<0.001**	**<0.001**	**<0.001**	**<0.001**	**<0.001**
Nitrogen (N)	**<0.001**	**<0.001**	**<0.001**	**<0.001**	0.376	0.054
Interactions between S and N	**<0.001**	**<0.001**	**<0.001**	0.169	0.461	0.390

Significant results are shown in bold.

**Figure 1:**
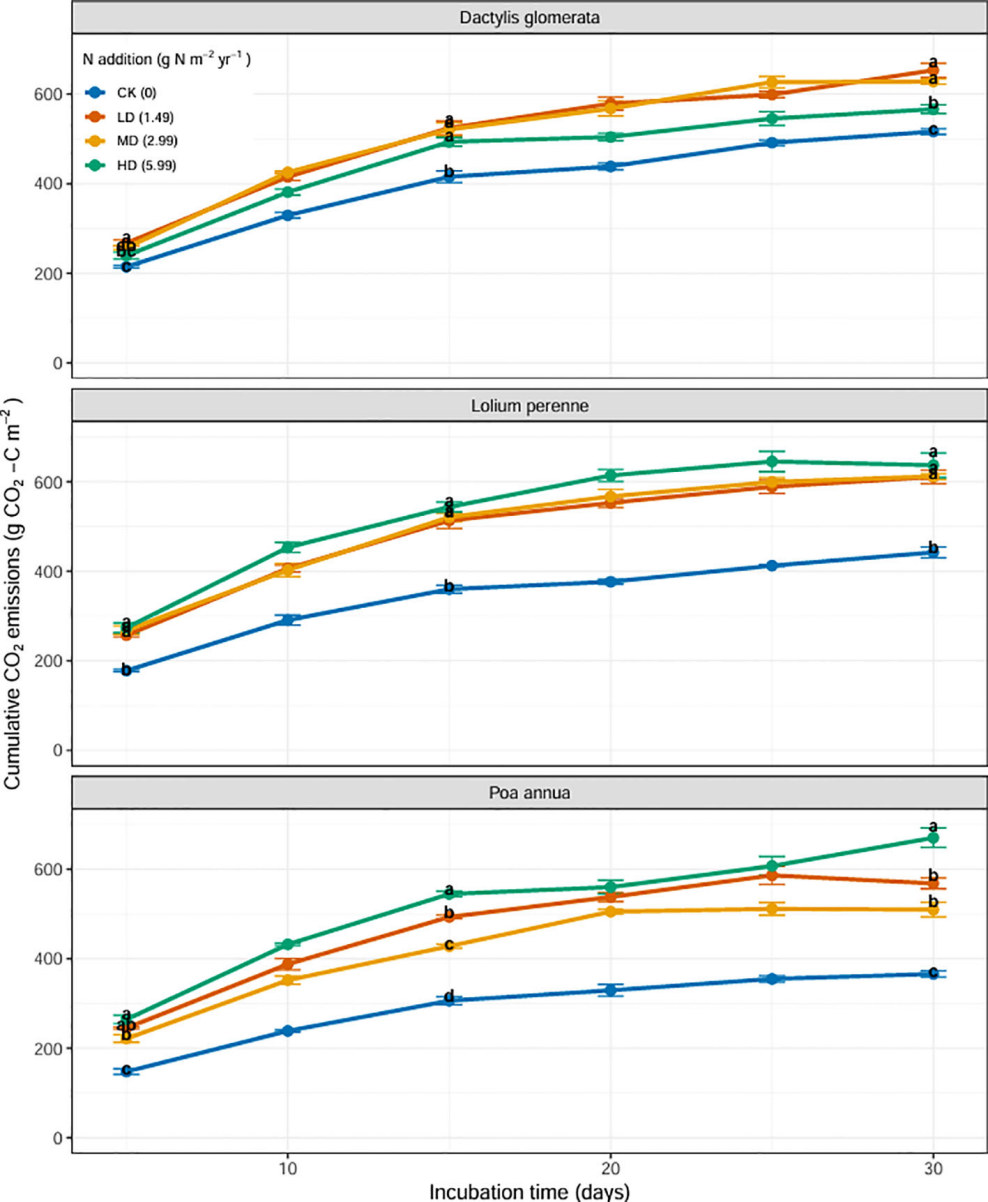
Cumulative CO_2_ efflux during litter decomposition under different nitrogen addition levels after 30 d of incubation. Different lowercase letters indicate statistically significant differences among the incubation time at the *p* < 0.05 level.

At each sampling interval, decomposition rates differed significantly among species (*P<* 0.001) for all intervals (**Figure 1**). *D. glomerata* consistently exhibited the highest decomposition rates, followed by *L. perenne*, while *P. annua* showed the lowest rates. For example, at day 5, decomposition rates were 1.8-fold higher for *D. glomerata* compared to P. annua in CK treatments. This pattern persisted throughout the incubation period, reflecting the influence of initial litter quality on decomposability. Nitrogen addition significantly accelerated decomposition for all species during the early incubation period (days 5–15, *P<* 0.001), but effects became non-significant at day 25 (*P* = 0.376) and marginally non-significant at day 30 (*P* = 0.054) (**Table 3**). The magnitude of N stimulation varied with litter quality, with low-quality litter (*P. annua*) showing greater relative acceleration than high-quality litter (*D. glomerata*). For instance, by day 5, decomposition rates of *P. annua* increased by 68% from CK to HD treatments, whereas *D. glomerata* showed only a 31% increase over the same N gradient. Significant interactions between species identity and N addition were detected during early decomposition stages (days 5-15, *P<* 0.001), but not during later stages (days 20-30, *P* > 0.05) (**Table 3**). This indicates that the stimulatory effect of N addition on decomposition is both species- and stage-dependent, with strongest interactive effects during the initial phase of labile C mineralization.

### 3.4 Decomposition rate constants (k values)

**Figure 2:**
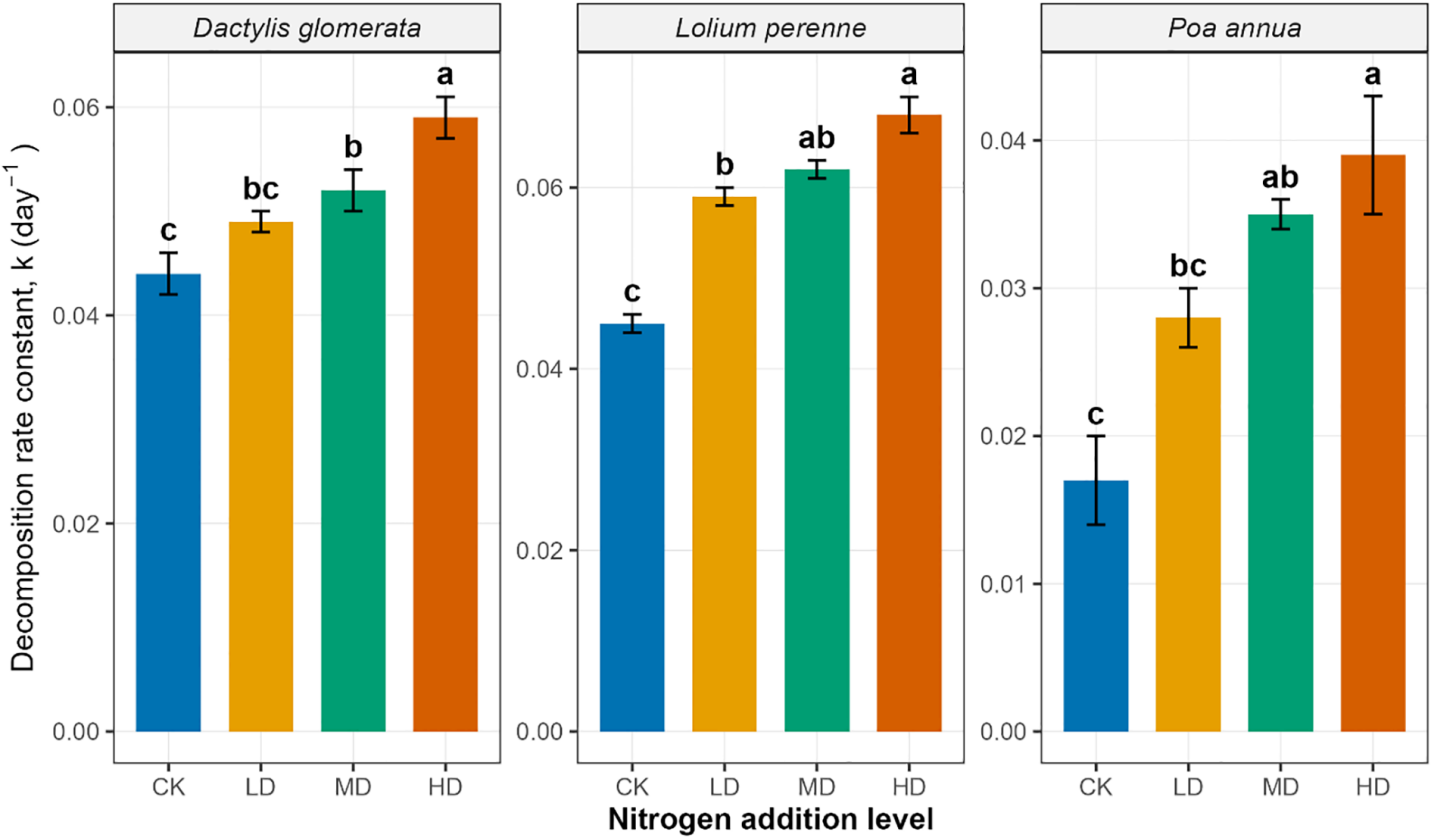
Effect of nitrogen addition on litter decomposition rate constant (k, day^-1^) after 30 days of decomposition in the incubation. Different lowercase letters indicate statistically significant differences among the nitrogen addition level at the *p* < 0.05 level.

First-order kinetic models provided excellent fits to cumulative CO_2_ efflux data for all treatment combinations, with (R^2^) values ranging from 0.978 to 0.998 (**Table 4**, **Figure 2**). Estimated decomposition rate constants (k) varied substantially across treatments, ranging from 0.017 day^-1^ (*P. annua* + CK) to 0.068 day^-1^ (*L. perenne* + HD).

**Table 4:** Litter decay constants (k, day^-1^) during litter decomposition in soils with different N addition after 30 days of decomposition in the incubation.

Treatments	k (day^-1^)	*S.E.*	*t* ratio	*P* value	*R* ^2^
*P. annua L.* + CK	0.017	0.003	9.44	**<0.001**	0.978
*L. perennel L.* + CK	0.045	0.001	8.65	**<0.001**	0.991
*D. glomerata* + CK	0.044	0.002	9.49	**<0.001**	0.998
*P. annua L.* + LD	0.028	0.002	10.24	**<0.001**	0.991
*L. perennel L.* + LD	0.059	0.001	8.95	**<0.001**	0.988
*D. glomerata* + LD	0.049	0.001	12.19	**<0.001**	0.998
*P. annua L.* + MD	0.035	0.001	16.47	**<0.001**	0.992
*L. perennel L.* + MD	0.062	0.001	13.65	**<0.001**	0.982
*D. glomerata* + MD	0.052	0.002	9.69	**<0.001**	0.998
*P. annua L.* + HD	0.039	0.004	19.8	**<0.001**	0.99
*L. perennel L.* + HD	0.068	0.002	18.35	**<0.001**	0.998
*D. glomerata* + HD	0.059	0.002	19.68	**<0.001**	0.998

Significant results are shown in bold.

Across N treatments, *P. annua* consistently exhibited the lowest (k) values (mean 0.030 day^-1^), *L. perenne* the highest (mean 0.059 day^-1^), and *D. glomerata* intermediate (mean 0.051 day^-1^). These differences were statistically significant for all pairwise comparisons (*P<* 0.001), confirming that litter quality strongly influences decomposition kinetics. For each species, k values increased progressively with N addition rate. For *P. annua*, k increased by 129% from CK (0.017 day^-1^) to HD (0.039 day^-1^), for *L. perenne*, by 51% from 0.045 to 0.068 day^-1^, and for *D. glomerata*, by 34% from 0.044 to 0.059 day^-1^. These patterns demonstrate that N addition accelerates decomposition, with the magnitude of stimulation being greatest for the lowest-quality litter.

### 3.5 Cumulative CO_2_ efflux

Cumulative CO_2_ efflux over 30 days incubation period ranged from 358.04 g CO_2_-C m^-2^ (*P. annua* + CK) to 665.09 g CO_2_-C m^-2^ (*L. perenne* + MD) (**Figure 2**; **Supporting Information Supplementary Table 1**). Species identity and N addition both significantly influenced cumulative efflux, with patterns generally mirroring those observed for decomposition rates. For a given N treatment, cumulative efflux followed the order: *P. annua*< *D. glomerata*< *L. perenne*. This ordering differed slightly from the pattern of k values, where *D. glomerata* and *L. perenne* were more similar, reflecting differences in potentially mineralizable C pools (C_0_) among species. For each species, cumulative efflux increased with N addition rate. The greatest relative increase was observed for *P. annua*, where efflux increased by 64% from CK to HD treatments, compared to 39% for *L. perenne* and 28% for *D. glomerata*. This pattern again supports the conclusion that N addition exerts stronger effects on decomposition of low-quality litter. The fitted first-order models captured cumulative efflux dynamics with high precision ((R^2^ > 0.98) for all treatments) (**Supporting Information Supplementary Table 2**). Estimated potentially mineralizable C pools (C_0_) varied among treatments but showed less consistent responses to N addition than (k) values, suggesting that N addition primarily affects decomposition rates rather than total mineralizable C.

### 3.6 Relationships between litter quality and decomposition

Pearson correlation analysis revealed strong relationships between initial litter quality parameters and decomposition kinetics (**Table 5**). Across all treatments and incubation intervals: N content showed significant positive correlations with decomposition rates during early stages (days 5-10, *r* = 0.536-0.687, *P<* 0.05) and negative correlations during later stages (days 25-30, *r* = -0.642, *P<* 0.01), reflecting a shift from N-controlled labile decomposition to C-controlled recalcitrant decomposition. C:N ratio exhibited negative correlations with decomposition rates in early stages (days 5-10, *r* = -0.510 to -0.625, *P<* 0.05) and positive correlations in later stages (days 25-30, *r* = 0.782, *P<* 0.01), mirroring the pattern observed for N content. N:P ratio showed consistently positive correlations with decomposition rates across most intervals, with strongest correlations in later stages (days 25-30, *r* = 0.983, *P* < 0.01). These patterns suggest that N availability (reflected in N content and C:N ratio) is the primary control during early decomposition, while P availability (reflected in N:P ratio) becomes increasingly important as decomposition proceeds.

**Table 5:** Pearson correlation coefficients between initial litter quality parameters and six litter decay constants (k, day^-1^).

K of each incubations periods	*N*_litter_	*P*_litter_	*C*_litter_ to *N*_litter_ ratio	*N*_litter_ to *P*_litter_ ratio
k_I_	0.687**	0.266	-0.625**	0.522
k_II_	0.536*	0.258	-0.510*	0.671
k_III_	0.294	0.034	0.138	-0.181
k_IV_	-0.006	-0.423	-0.276	0.036
k_V_	-0.369	-0.554*	0.466*	0.980**
k_VI_	-0.642**	-0.761**	0.782**	0.983**

Six ncubations periods (I,II, III, IV, V and VI). Asterisks indicate degree of significance (*P< 0.05; **P < 0.01).

## 4 Discussion

### 4.1 Litter quality as a primary determinant of decomposition rates

Our results provide strong support for the first hypothesis that litter quality, particularly initial N content and C:N ratio, exerts a dominant control on decomposition rates in subtropical grassland ecosystems. *D. glomerata* litter, with higher N content (16.3 mg·g^-1^) and lower C:N ratio (28.5), decomposed significantly faster than *P. annua* litter, which had lower N content (12.5 mg·g^-1^) and higher C:N ratio (36.8). This aligns with the classical paradigm that high-quality litter provides a more balanced supply of C and N to microbial communities (Melillo et al., 1982; Cornwell et al., 2008; Makkonen et al., 2012; Zhang et al., 2008). Moreover, recent work has demonstrated that fast-decomposing litter can enhance soil carbon storage through efficient microbial processing, challenging the traditional view that only recalcitrant litter contributes to long-term carbon pools (Garcia-Palacios et al., 2021). Stoichiometric constraints on decomposition under elevated N deposition further highlight the importance of initial litter N:P ratios (Png et al., 2023).

The mechanistic basis for this pattern lies in the nutritional requirements of decomposer microorganisms (Manzoni et al., 2010). High-quality litter, with its higher N content and lower C:N ratio, provides a more balanced supply of C and N to microbial communities, reducing the need for microbes to invest in N acquisition and allowing more efficient allocation of resources to C mineralization (Hobbie, 2008; Mooshammer et al., 2012). Conversely, low-quality litter with high C:N ratio creates an N-limited environment that constrains microbial growth and activity, slowing decomposition (Hodge et al., 2000; Craine et al., 2007).

The strong negative correlations we observed between initial C:N ratio and decomposition rates during early incubation stages (*r* = -0.510 to -0.625, *P<* 0.05) are consistent with this interpretation. Notably, these correlations weakened or reversed in later stages, suggesting that the relative importance of N availability declines as decomposition proceeds and the remaining material becomes enriched in recalcitrant compounds (Berg and McClaugherty, 2014). This stage-dependent pattern highlights the importance of considering decomposition dynamics over time rather than relying solely on single-time-point measurements.

### 4.2 Nitrogen addition accelerates decomposition through alleviation of microbial N limitation

Our results also support the second hypothesis that N addition accelerates litter decomposition, with greater stimulation for low-quality litter. Importantly, because all litter was collected from unfertilized reference areas, our experimental design specifically tests the soil legacy effects of four years of N addition—including increased inorganic N pools and reduced pH—on decomposition of standardized litter. This design does not allow us to directly test whether N addition alters litter quality. For *P. annua* (lowest initial N content), k increased by 129% from CK (0.017 day^-1^) to HD (0.039 day^-1^), whereas for *D. glomerata* (highest initial N content), the increase was only 34% (from 0.044 to 0.059 day^-1^). This pattern is consistent with the concept of “microbial N mining” (Craine et al., 2007; Moorhead and Sinsabaugh, 2006), whereby microorganisms decomposing low-quality litter are strongly limited by N availability compared to the higher N treatments, even though the region receives moderate ambient N deposition (2.87 g N m^-2^ yr^-1^; Wang et al., 2016). Thus, the “N limitation” we refer to is a relative constraint imposed by the stoichiometric imbalance of the litter, not an absolute N deficiency of the ecosystem. This relative limitation explains why low-quality litter responds more dramatically to the enhanced soil N pools resulting from long-term N addition.

The mechanisms underlying N stimulation of decomposition are multifaceted. First, N addition directly alleviates microbial N limitation, enabling more rapid growth and metabolic activity (Hobbie, 2008; Knorr et al., 2005). Second, N addition can stimulate production of extracellular enzymes involved in C degradation, as shown in subtropical forests (Chen et al., 2021; Hou et al., 2021). Third, N addition may alter the composition and activity of decomposer communities, favoring faster-growing microbial taxa (Frey et al., 2004; Ramirez et al., 2012). Furthermore, the interactive effects of N deposition and litter quality on soil carbon cycling are mediated by shifts in microbial functional genes and enzyme stoichiometry (Guo et al., 2022; Tang et al., 2020).

However, the stimulatory effect was most pronounced during days 10–15 and diminished thereafter. This stage-dependent response reflects the fact that initial decomposition involves primarily labile C compounds (sugars, starches, proteins) that are strongly responsive to N availability, while later stages dominated by recalcitrant compounds may be less responsive (Berg and McClaugherty, 2014). Our results are consistent with a recent global meta-analysis showing that N addition effects on litter decomposition are strongest in grasslands and for low-quality litter, but that these effects diminish over longer experimental periods (Wang et al., 2025).

Several limitations of this study should be acknowledged. First, the 30-day incubation period captures only the initial, labile phase of decomposition; therefore, extrapolating these short-term results to long-term ecosystem carbon dynamics is not warranted. Second, the application of a first-order kinetic model to the 30-day data provides only a short-term approximation of decomposition kinetics. Consequently, the estimated potentially mineralizable carbon pools (C_0_) should not be interpreted as representing true long-term mineralization potentials. Third, and most critically, our experimental design does not allow us to separate the effects of nitrogen (N) addition on soil properties from its potential effects on litter chemistry, as all litter was collected from unfertilized reference areas. Future studies should employ litter grown under different N treatments to more rigorously test the proposed mechanism of altered litter quality. Fourth, our study was conducted under controlled laboratory conditions (constant 25 °C, optimal moisture) that do not reflect the temperature and moisture fluctuations typical of field environments. Additionally, it should be noted that the litter was ground into powder to ensure homogeneity and reproducibility, a common practice in controlled incubation studies. However, this mechanical disruption inevitably destroys the natural tissue structure. In natural decomposition, the gradual breakdown of physical structures by microbial extracellular enzymes is a critical rate-limiting step. As grinding potentially bypasses this structural deconstruction phase, the actual decomposition rates—or their relative differences—observed in the field may differ from our laboratory results. Caution is therefore necessary when extrapolating our findings to field conditions where litter structure remains intact.

## 5 Conclusion

This study investigated the soil legacy effects of long-term nitrogen (N) addition on litter decomposition in a subtropical grassland. Litter quality strongly influenced decomposition rates, with high-quality litter decomposing faster. N addition accelerated decomposition for all species, with the strongest effect on low-quality litter, supporting the microbial N mining hypothesis. However, because all litter was collected from unfertilized reference areas, our results specifically demonstrate soil legacy effects—i.e., N-induced changes in soil properties—rather than N-induced changes in litter quality. The stimulatory effect was most pronounced during early decomposition stages. We conclude that long-term N addition alters soil properties, which in turn accelerate the decomposition of subsequently added litter, leading to enhanced total CO_2_ release from the litter-soil microcosms. Future research should extend incubation periods to capture later decomposition stages, employ litter grown under differential N inputs to directly test the “altered litter quality” mechanism, and incorporate field-based measurements to validate our laboratory findings under ambient environmental conditions.

## Data Availability

The original contributions presented in the study are included in the article/**Supplementary Material**. Further inquiries can be directed to the corresponding author.
