# Supplementary material for: Soil legacy effects of long-term nitrogen addition on litter decomposition and soil CO2 efflux in a subtropical grassland: a short-term incubation study
Source: Front Plant Sci. 2026 Jun 15;17:1849132. doi: 10.3389/fpls.2026.1849132 (PMC13310747; doi:10.3389/fpls.2026.1849132)
Supplement: Supplementary file 1 [file Table1.docx]

**Table** **S1** The total of cumulative CO_2_ emissions from soils with different litter and N addition after 30 d of incubation

| Treatments | Cumulative CO_2_ emissions (g m^-2^) |
| --- | --- |
| *Poa annua L.* + CK | 358.04 |
| *Lolium perennel L.* + CK | 434.46 |
| *Dactylis glomerata* + CK | 496.97 |
| *Poa annua L.* + LD | 527.22 |
| *Lolium perennel L.* + LD | 617.68 |
| *Dactylis glomerata* + LD | 634.28 |
| *Poa annua L.* + MD | 645.49 |
| *Lolium perennel L.* + MD | 665.09 |
| *Dactylis glomerata* + MD | 566.31 |
| *Poa annua L.* + HD | 588.72 |
| *Lolium perennel L.* + HD | 603.85 |
| *Dactylis glomerata* + HD | 634.28 |

**Table S2** Models (equations) of cumulative CO_2_ emissions of soils with different litter types and N addition.

| Treatment | Models | R^2^ |
| --- | --- | --- |
| Litter of *Poa annua L.* + CK | C _m_= 896.2 (1 – e^-0.017t^) | 0.978 |
| Litter of *Poa annua L.* + LD | C _m_= 764.5 (1 – e^-0.028t^) | 0.991 |
| Litter of *Poa annua L.* + MD | C _m_= 548.3 (1 – e^-0.035t^) | 0.992 |
| Litter of *Poa annua L.* + HD | C _m_= 468.7 (1 – e^-0.039t^) | 0.990 |
| Litter of *Lolium perennel L.* + CK | C _m_= 546.5 (1 – e^-0.055t^) | 0.991 |
| Litter of *Lolium perennel L.* + LD | C _m_= 522.3 (1 – e^-0.059t^) | 0.988 |
| Litter of *Lolium perennel L.* + MD | C _m_= 508.7 (1 – e^-0.062t^) | 0.982 |
| Litter of *Lolium perennel L.* + HD | C _m_= 448.0 (1 – e^-0.068t^) | 0.998 |
| Litter of *Dactylis glomerata* + CK | C _m_= 646.9 (1 – e^-0.045t^) | 0.998 |
| Litter of *Dactylis glomerata* + LD | C _m_= 528.5 (1 – e^-0.049t^) | 0.998 |
| Litter of *Dactylis glomerata* + MD | C _m_= 510.3 (1 – e^-0.052t^) | 0.998 |
| Litter of *Dactylis glomerata* + HD | C _m_= 496.1 (1 – e^-0.059t^) | 0.998 |
